# High-Resolution Patterning of Organic Emitting-Layer by Using Inkjet Printing and Sublimation Transfer Process

**DOI:** 10.3390/nano12091611

**Published:** 2022-05-09

**Authors:** Jun Yeub Lee, Byeong-Kwon Ju, Kwan Hyun Cho

**Affiliations:** 1 Digital Transformation R&D Department, Korea Institute of Industrial Technology, Ansan-si 15588, Gyeonggi-do, Korea; june522@kitech.re.kr; 2 Display and Nanosystem Laboratory, School of Electrical Engineering, Korea University, Seoul 02841, Seoungbuk-gu, Korea

**Keywords:** inkjet printing, co-solvented ink, vacuum drying process, sublimation transfer process, high resolution patterning, ohnesorge number

## Abstract

We implemented ultra-high resolution patterns of 2822 pixels-per-inch (PPI) via an inkjet printing and vacuum drying process grafted onto a sublimation transfer process. Co-solvented ink with a 1:1 ratio of N,N-dimethylformamide (DMF) to ortho-dichlrorobenzene (oDCB) was used, and the inkjet driving waveform was optimized via analysis of Ohnesorge (Oh)—Reynolds (Re) numbers. Inkjet printing conditions on the donor substrate with 2822 PPI microchannels were investigated in detail according to the drop space and line space. Most sublimation transferred patterns have porous surfaces under drying conditions in an air atmosphere. Unlike the spin-coating process, the drying process of inkjet-printed films on the microchannel has a great effect on the sublimation of transferred thin film. Therefore, to control the morphology, we carefully investigated the drying process of the inkjet-printed inks in the microchannel. Using a vacuum drying process to control the morphology of inkjet-printed films, line patterns of 2822 PPI resolution having a root-mean-square (RMS) roughness of 1.331 nm without voids were successfully fabricated.

## 1. Introduction

Inkjet printing technologies for pixel composition of organic light-emitting diodes (OLED) have been widely investigated in recent years [1,2,3,4]. Due to its simplicity, inkjet printing is a solution-processed technology that has attracted attention to enable a variety of applications [5,6,7]. Additionally, high-efficiency and ultra-high-resolution displays are in the spotlight to provide a main display for extended reality (XR) beyond mixed reality (MR) [8,9,10,11,12]. However, conventional inkjet printing technology has crucial limitations in fabricating high-resolution patterns [13,14,15]. Pixel defining methods such as color filter technology, fine metal mask, and photolithography still have problems. The color filter method has a decrease in brightness due to the device structure [16]. Fine metal mask and photolithography methods, which are direct patterning methods, also accompany a shadow effect from the distance between substrates [17,18] and chemical limitations such as damage to organic materials caused by process characteristics [19,20]. In our previous research, we showed the possibility of fabricating high-resolution patterns with high stability through solution and evaporation hybrid technology [4,21,22]. However, in our previous work, a spin-coating process was used to fill ink into the microchannel [21,22], or an inkjet-printing process including a non-sublimable additive to conventional inks was used [4]. The spin-coating process is difficult to apply to the production of large-area glass substrates, and the additives can reduce the device performance of OLEDs. Our color patterning method also has some disadvantages, such as increase in process complexity, possibility of pattern blurring. Additional processes of selective surface treat-ment of donor substrate, sublimation transfer process in the vacuum chamber, and vac-uum drying processes after inkjet printing are required for the fabrication of emitting-layer (EML) patterns. These additional processes can increase the process complexity. However, the fabrication of high-resolution FMM is very difficult in the FMM method [17], and pro-cess complexity and new material development are required in the photolithography method [19]. In addition, the problem of pattern blurring becomes more serious as the pat-tern resolution increases. However, the pattern blurring can be prevented through the complete contact between the donor and target substrates during the sublimation transfer process. In this paper, we present high-resolution patterning of an organic emitting layer (EML) using inkjet printing and sublimation transfer technology through conventional inks without additives, circumventing limitations such as the shadow effect of the fine metal mask method and developing new chemicals to address chemical damage to organic material, a high-resolution patterning method has been proposed. Inkjet printing can also construct patterns on large area substrates [23]. This suggests that the inkjet printing technology could join the ranks of the direct patterning method for high-resolution patterning above 2800 PPI through the work in this study. First, based on an analysis of Oh and Re values, we carefully optimized the inkjet driving waveforms using co-solvented inks of oDCB and DMF to enhance drop precision onto the 2822 PPI microchannel. In addition, to evenly fill the EML inks over a large area of the donor substrate, we systematically analyzed the ink filling properties through variations of the drop space and line space. Meanwhile, the drying mechanism in the inkjet printing process is very different from those of the spin-coating process, and morphology control is very difficult due to the complicated drying kinetics [13,24,25,26]. Furthermore, we found that the inkjet-printed donor substrate, which, according to the dry condition, influences the sublimation transferred pattern. Therefore, we carefully controlled the morphology of the inkjet-printed EML films on the microchannel during the drying process in vacuum conditions. Through optimization of these inkjet and vacuum drying conditions, we think the fabrica-tion of high-resolution patterns were performed by synergic effect [27]. Finally, the high-resolution 2822 PPI patterns of EML obtained using the inkjet printing process grafted onto the sublimation transfer process were implemented via optimization of the drying condition.

## 2. Materials and Methods

### 2.1. Preparation of Materials and Co-Solvented Ink

The EML materials of 4,4′-Bis(9-carbazolyl)-1,1′-biphenyl (CBP) and bis(2-methyldibenzo[f,h]quinoxaline)(acetylacetonate) iridium(III) (Ir(MDQ)_2_acac) were purchased from OSM Corp., Korea. The solutions of co-solvented ink were prepared using mixtures of the following solvents; Chlorobenzene (CB) (99.8%, anhydrous, Sigma Aldrich, Seoul, Korea), ortho-dichlorobenzene (oDCB) (99%, anhydrous, Sigma Aldrich, Seoul, Korea), and N,N-dimethylformamide (DMF) (99.8%, anhydrous, Sigma Aldrich, Seoul, Korea). The solution of the co-solvented inks was mixed at a 2:1 volume ratio of CB:oDCB and 1:1 ratio of oDCB:DMF. The materials for EML have Ir(MDQ)_2_acac serving as the dopant and CBP serving as the host. For the red EML ink, the mixtures of CBP and 10 wt. % Ir(MDQ)_2_acac were dissolved in co-solvented ink at a concentration of 7.5 mL/mg during an 8 h stirring.

### 2.2. Selective Surface Treatment of Donor Substrate

The donor substrate has microchannel and bank structures on the light to heat conversion (LTHC) layer. The substrate was cleaned with acetone, isopropanol, and chloroform in a sonication bath for one and a half hours to completely remove organics in the microchannel. The piranha solution was prepared at a mix ratio of 2:1 of sulfuric acid (95%, Samchun, Seoul, Korea) and hydrogen peroxide (34.5%, Samchun, Seoul, Korea). 1.4 vol% of octadecyltrichlorosilane (OTS) (95%, Acros Organics, Seoul, Korea) was used, dissolved in toluene (99.8%, Samchun, Seoul, Korea) for selective surface treatment. The pretreated with piranha solution, to hydroxylate the substrate for easy combination with OTS [28]. Subsequently, the substrate was immersed in toluene solution for 30 min, rinsed in pure toluene, and completely dried with N_2_. Following the drying of the substrate, a short UV/Ozone treatment (AH-1700, AHTECH LTS, Korea) was made to change the microchannel and the bank into hydrophilic and hydrophobic, respectively [22,29,30].

### 2.3. Inkjet Printing into Microchannel of Donor Substrate

Red co-solvented ink was inkjet-printed using the Inkjet printing system (Marvel Engineering, Seongnam, Korea) with a dimatix-samba cartridge (Fujifilm Co., Tokyo, Japan) under an N_2_ atmosphere. The applied rising and falling times for the waveform were 2.2 μs and 2.8 μs, respectively. The pulse time of the applied voltage on the waveform was in a range between 2.8 μs and 4.3 μs. The applied voltage was set in a range between 22 V and 26.5 V, enabling inkjet printing of co-solvented ink with DMF and oDCB. The speed of the droplet was approximately 3.2 m/s, and the distance between the substrate and the nozzle was 300 μm. Subsequently, inkjet printing was performed on the donor substrate by variation of the line and drop spaces. The line spaces, determined by the X-axis pitch, were 9 μm, 18 μm, and 27 μm, because the donor substrate had a line pitch of 9 μm. Drop spaces, which are the Y-axis pitch, were 20 μm, 25 μm, and 33 μm in our experiment.

### 2.4. Drying and Sublimation Transfer Process

A variety of conditions were employed to inkjet-print the EML pattern with the co-solvented red ink onto the donor substrate and dried to get rid of residual solvent and solidify the pattern. A high vacuum drying system (Samhan Vacuum, Gwangmyeong, Korea) was used, which can make a high vacuum condition within a short time for the drying process. The drying conditions of the inkjet-printed patterns were room temperature, 30 °C, 50 °C, and 80 °C, all held for 20 min in the vacuum chamber under a 5 × 10^−5^ Torr atmosphere. For the sublimation transfer process, a xenon flash lamp system (XF 15200LCW, Unilam Co., Ltd., Ulsan, Korea) was used. The donor substrate was loaded upside down on the target substrate in a high vacuum chamber under an 8 × 10^−7^ Torr atmosphere. The backside of the donor substrate was exposed to intense pulse light (IPL) at an energy of 7.8 J/cm^2^.

### 2.5. Characteristics

A VHX-6000 digital microscope (Keyence, Japan) and an NX10 system (Park Systems Co., Suwon, Korea) were used to obtain optical images of the substrate and the surface morphology of the EML pattern, respectively. A UHR FE-SEM (Hitachi High-Technologies Corp., Tokyo, Japan) was used to obtain a scanning electron microscope image of the donor substrate surface and cross-section image. A FluoroMate FS-2 spectrometer (Scinco Co., Seoul, Korea) was used to obtain the photoluminescence (PL) spectra.

## 3. Results and Discussion

### 3.1. Sequence of Patterning Process

**Figure 1:**
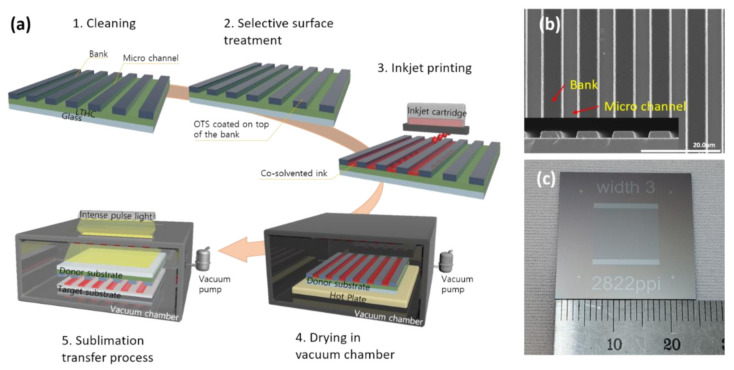
(**a**) Sequential schematic diagram of the patterning process by using inkjet printing grafted onto the sublimation transfer process. (**b**) SEM images of the LTHC substrate’s top and side views and (**c**) a picture of the LTHC donor substrate.

Ultra-high resolution patterning with inkjet printing grafted onto the sublimation transfer process has hardly been reported yet. Figure 1a provides a sequential schematic diagram of the patterning process in our experiment through which inkjet printing was grafted onto the sublimation transfer process. In previous research [21,22,31], high-resolution patterns with a pitch of about 14 μm were successfully obtained by spin-coating and sublimation transfer processes. To use inkjet printing to implement the ultra-high resolution pattern, the donor substrate was designed with a 9 μm pitch and a 3 μm width of microchannel; actually fabricated, the pitch and the microchannel width were approximately 9 μm and 4 μm, respectively. The microchannel and bank, structured on the donor substrate, are shown in the SEM image in Figure 1b; an overall picture of the donor substrate is provided in Figure 1c. After the cleaning process of the donor substrate, a selective surface was treated with OTS to obtain a hydrophilic microchannel and a hydrophobic bank. By controlling wettability, the top of the bank coated with OTS induced the inkjet-printed ink into the microchannel. The donor substrate filled with ink was loaded for a specific time into the vacuum chamber of the drying equipment for drying and solidification of the ink pattern. The dried donor substrate was then loaded upside down, together with the target substrate, in the vacuum chamber of the IPL system. The sublimation transfer process was employed using an IPL exposure that transfers the pattern onto the target substrate.

### 3.2. Optimization of Inkjet Condition of Co-Solvented Ink

The properties of the ink are important in the inkjet printing process [13,14]. To control the properties, co-solvented ink was prepared, including CB, oDCB, and DMF; the host and dopant materials were dissolved in this co-solvented ink. The single solvent properties for the production of the co-solvented ink are provided in Table S1. In previous studies, co-solvented ink with a 2:1 ratio of CB to oDCB was prepared to compare inkjet-printing conditions [21]. In previous studies, co-solvented ink was used in the spin-coating process to fill ink into the microchannel. It is very important to create a stable ink-jetting condition for precise inkjet printing on the 2822 PPI microchannel. Therefore, another co-solvented ink was prepared at a 1:1 ratio of oDCB to DMF. To compare the inkjet-printing conditions of the prepared inks, the following dimensionless numbers were used [32,33]. These dimensionless numbers are expressed as follows:

(1) $$\mathrm{Weber}\;\mathrm{Number}\;(\mathrm{We})=\frac{v^{2}\rho d}{\gamma }$$

(2) Reynolds Number (Re)=vρdμ

(3) $$\mathrm{Ohnesorge}\;\mathrm{Number}\;(\mathrm{Oh})=\frac{\sqrt{We}}{Re}=\frac{\mu }{\sqrt{\gamma \rho d}}$$

**Figure 2:**
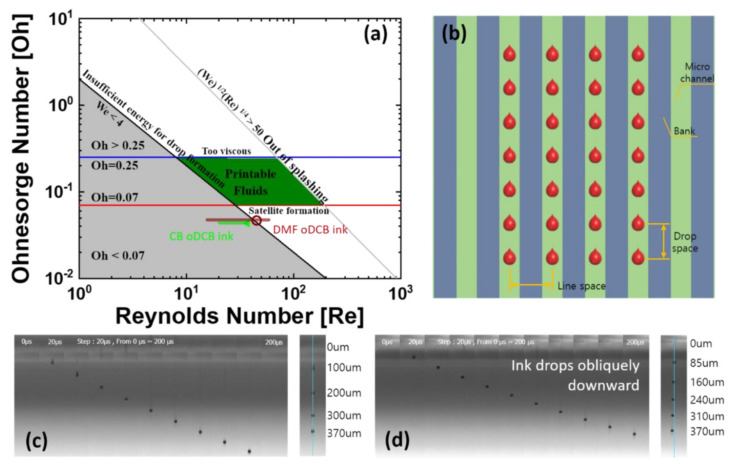
(**a**) Ohnesorge map of the area of the printable fluid and dimensionless numbers of produced co-solvented inks (**b**) schematic diagram of inkjet-printed drop positions onto microchannel. Ink drop images with co-solvented (**c**) with DMF and oDCB ink, and (**d**) with CB and oDCB ink.

The value v is the droplet speed, ρ is the density of the ink, d is the diameter of the nozzle, γ is the viscosity of the ink, and μ is the surface tension of the ink. The value of We can be used to determine which parameter is more important between the fluid’s inertia and its surface tension. If the value of We is less than 4, or if (We)^1/2^(Re)^1/4^ is larger than 50, the ink has insufficient or excessive energy for droplet formation. The value Re is the ratio of inertial to viscous forces in the relative movement between different velocities of fluids conditions. Oh represents the square root of We divided by Re [34]. If Oh is less than 0.07 or larger than 0.25, ink for the inkjet printing generates satellites of droplets or lengthened droplet tails, respectively [32]. Figure 2a shows the area of the printable fluid on an Ohnesorge map and indicates dimensionless numbers of produced co-solvented inks. On the map, for the co-solvented ink with CB and oDCB, the marked ink-jettable area is green in color; this area has an Oh of 0.0431, a Re range from 24.14 to 49.87, and a We range from 1.08 to 4.61. The co-solvented ink with DMF and oDCB, marked as the ink-jettable area in brown color on the map, had an Oh of 0.0467, a Re range from 15.75 to 59.44, and a We range from 0.54 to 7.70. These dimensionless numbers were calculated by measured drop speed according to the pulse time and voltage of the waveform, marked with numbers in Figure S1 and with the properties of the solvents given in Table S1. The shadowed orange area in Figure S1 represents the inkjet printable condition of the co-solvented inks. The condition of the co-solvented ink was green (CB and oDCB ink; We: 2.53, Re: 36.91, Oh: 0.0431) and brown (DMF and oDCB ink; We: 4.42, Re: 45.04, Oh: 0.0467) dots on the Ohnesorge map were used in the experiment. For the same inkjet system and ink condition, only the drop speed is an effective variable for controlling the waveform because it can affect the Reynolds number and the Weber number. Unfortunately, the dimensionless numbers of the co-sovented inks did not intersect with the printable fluids regions on the Ohnesorge map. However, co-solvented ink with DMF and oDCB had a range closer to the printable fluids region. The results in Figure 2c show ink drop images of DMF and oDCB co-solvented ink, which fell straight, without splashing or shifting from the original trajectory, up to 4.2 m/s drop speed. On the other hand, Figure 2d shows ink drop images with CB and oDCB co-solvented ink, which fell obliquely downward even at 2.1 m/s drop speed. The ink drop shifted 8.57 μm from its original drop trajectory at a distance of 300 μm from the nozzle. This is enough to have an effect on the ink filling condition in the next microchannel. The ink drop should be straight down because it should allow printing in an accurate position on the microchannel to control ink filling and to make a more precise pattern. By considering the dimensionless numbers and ink drop images, co-solvent ink with DMF and oDCB was found to have more congruence with the inkjet-printing process than the ink with CB and oDCB.

### 3.3. Ink Filling Properties According to Variation of Drop and Line Space

**Figure 3:**
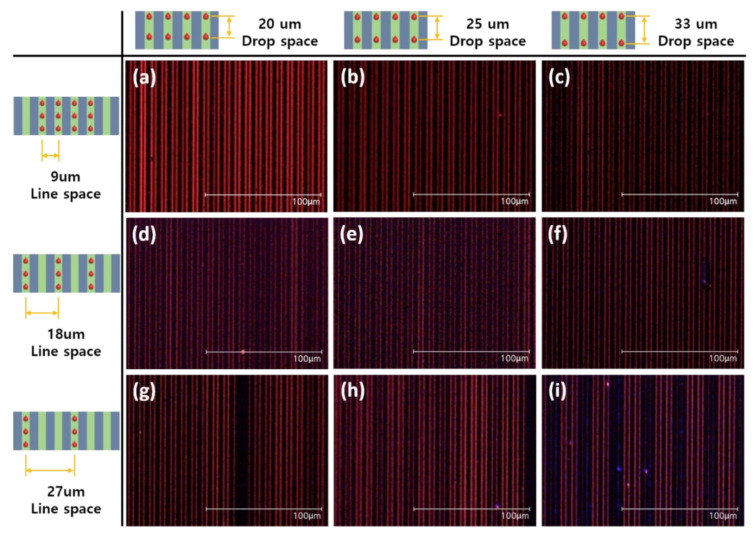
OM images of inkjet-printed onto the microchannel on the donor substrate with UV exposure according to the drop and line space. Inkjet-printed with (**a**–**c**) 9 μm, (**d**–**f**) 18 μm, and (**g**–**i**) 27 μm for line space, and (**a**,**d**,**g**) 20 μm, (**b**,**e**,**h**) 25 μm, (**c**,**f**,**i**) and 33 μm for drop space.

Figure 2b provides a schematic diagram of the inkjet-printed drop position on the microchannel. The donor substrate had a 9 μm pitch and an approximately 4 μm width of the microchannel. The droplet size of the printed ink was larger than the width of the microchannel. The selective surface-treated donor substrate-induced ink droplets into the microchannel. So, the line space of the printing condition used multiples of the pitch of the donor substrate to determine an optimized value for uniform ink filling into the microchannel. The drop space of the printing condition was also needed to determine the optimized condition, and so values were tried of 20 μm, 25 μm, and 33 μm. Inkjet-printed donor substrates were dried in an air atmosphere at 100 °C for 5 min. In the case of the longer time drying process, the inkjet-printed patterns were broken, and the host and dopant materials were separated from the microchannel even at the lower temperature that we used. Figure S2a provides a UV exposed optical microscope (OM) image of the host and dopant material separated from the microchannel in the drying process at a high temperature, which partially emits blue. During the drying at a high temperature in an air atmosphere, the inkjet-printed pattern solidified from the surface to the inside. The vaporized residual solvents separate the emitting materials from the microchannel, as shown in the schematic diagram provided in Figure S2b [35,36]. Figure 3 shows UV exposed OM images of the inkjet-printed co-solvented ink in the microchannel. Inkjet-printed conditions were (Figure 3a–c) 9 μm, (Figure 3d–f) 18 μm, and (Figure 3g–i) 27 μm for line space and (Figure 3a,d,g) 20 μm, (Figure 3b,e,h) 25 μm, and (Figure 3c,f,i) 33 μm for drop space, as shown in Figure 3. Inkjet-printed pattern morphologies in the microchannel were strongly affected by the number of ink droplets per unit area. As a result of widening the line space from 9 μm to 27 μm and the drop space from 20 μm to 33 μm, the amount of EML ink filled into the microchannel decreased, and the morphology of the patterns became non-uniform.

**Figure 4:**
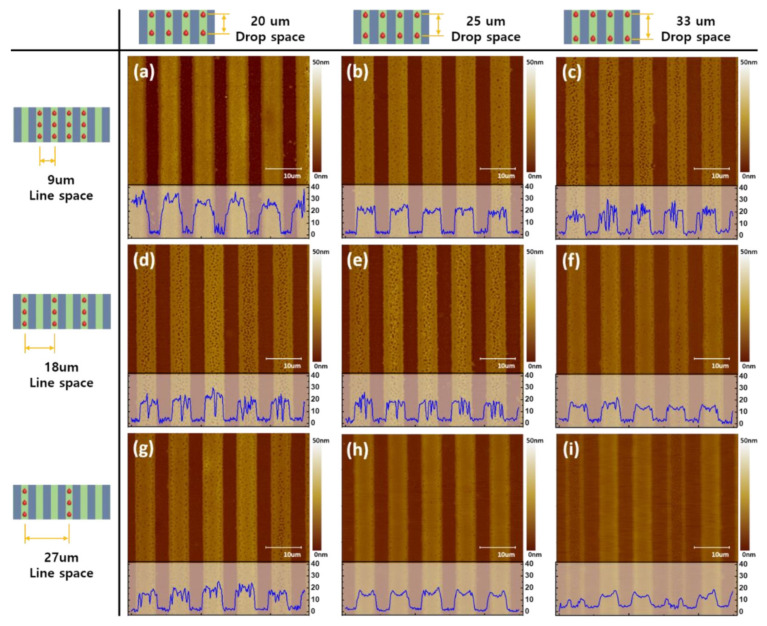
AFM images of sublimation transferred patterns according to the drop and line space. Inkjet-printed with (**a**–**c**) 9 μm, (**d**–**f**) 18 μm, and (**g**–**i**) 27 μm for line space, and (**a**,**d**,**g**) 20 μm, (**b**,**e**,**h**) 25 μm, (**c**,**f**,**i**) and 33 μm for drop space. Insets are cross-sectional data, respectively.

To investigate the morphology of patterns in detail, atomic force microscope (AFM) analysis was used. The results are shown in AFM images and cross-sectional graphs of the sublimation transferred patterns in Figure 4. The conditions were, as shown in Figure 4 a–c 9 μm, Figure 4d–f 18 μm, and Figure 4g–i 27 μm for line space and Figure 4a,d,g 20 μm, Figure 4b,e,h 25 μm, and Figure 4c,f,i 33 μm for drop space. The 9 μm line space and 25 μm drop space condition had the lowest roughness on the top of the pattern and was very uniform compared to surfaces formed using other conditions. The results for 18 μm and 27 μm of line space show overflow to the next line; this caused the ink to insufficiently fill into the microchannel. The inkjet printing condition of 9 μm line space and 20 μm drop space leads to a thick pattern and retains overflow traces on the bank surfaces. As a result, most of the sublimation transferred pattern has a porous surface. These porous surfaces were assumed to be caused by the sublimation transfer process and the non-uniformity of the dried EML ink in the microchannel. While the spin-coating process leads to a relatively uniformly dried film due to the centrifugal force caused by the rotation, the dried films fabricated by the inkjet printing process tend to induce non-uniformity due to the capillary and the Marangoni flow during the drying process [36,37,38].

### 3.4. High-Resolution EML Patterning by Optimization of Vacuum Drying Condition

**Figure 5:**
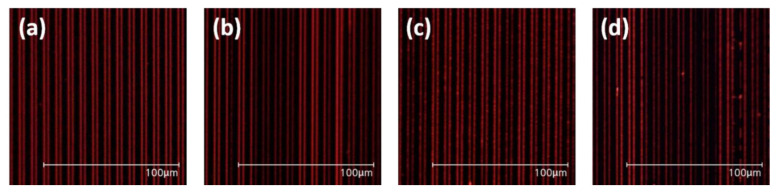
UV exposed OM images of inkjet-printed onto the microchannel of the donor substrate after drying (**a**) at room temperature, (**b**) 30 °C, (**c**) 50 °C, and (**d**) 80 °C in a vacuum chamber.

We carefully investigated the morphology of inkjet-printed EML films depending on the presence of heating during the drying process under vacuum conditions. The vacuum drying process rapidly reduces the pressure in the vacuum chamber, which causes a reduction in the boiling point, enabling the evaporation of solvents in the microchannel in a short time without damaging the organic materials [39]. The enhanced solvent evaporation rate indicated restraining the coffee ring effect [40]. In addition, the residual solvent can evaporate and dry faster from the microchannel via a phenomenon known as bumping [41,42]. As mentioned in the previous paragraph, drying at a high temperature in the air atmosphere causes breakage and separation of the emitting materials in the microchannel. Therefore, the inkjet-printed pattern in the microchannel under drying conditions in the vacuum chamber was investigated. The method of vacuum drying involves drying the substrate in a vacuum chamber under 5 × 10^−5^ Torr. The EML patterns were inkjet-printed into the microchannel under 9 μm line space and 25 μm drop space conditions and dried under temperature variation in the vacuum chamber. UV exposed OM images of the donor substrate inkjet-printed pattern are shown in Figure 5, including substrates dried: (a) at room temperature, (b) at 30 °C, (c) at 50 °C, and (d) at 80 °C. Drying via heat in the vacuum chamber, the inkjet-printed pattern in the microchannel rather had a degraded uniformity due to the fast evaporation. However, compared to the case of using an air atmosphere, the pattern dried with heat in the vacuum chamber was not broken or separated from the microchannel.

**Figure 6:**
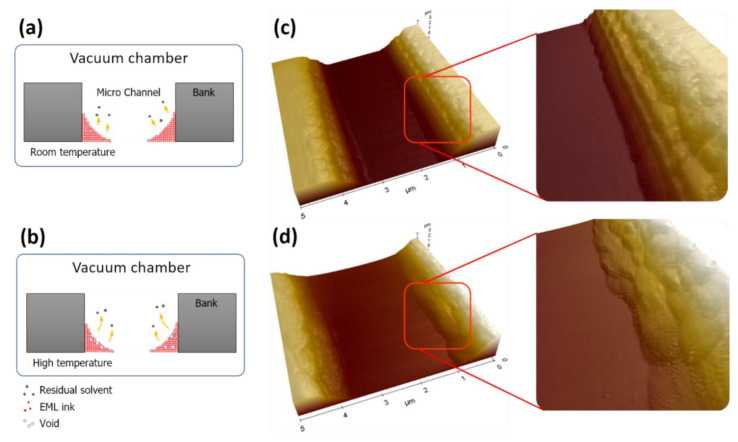
Schematic diagram of the residual solvent behavior and ink solidifying state (**a**) at room temperature and (**b**) at high temperature in the vacuum drying process. The AFM images of the solidified ink in the microchannel at (**c**) room temperature and (**d**) high temperature according to the vacuum drying process.

On the other hand, the drying condition at room temperature led to the uniformity of the pattern inkjet-printed in the microchannel. Figure 6a,b shows schematic diagrams of the residual solvent behavior during solidification at room temperature and at high temperature, respectively, during vacuum drying. During solidification at room temperature, the residual solvent rapidly came out due to a reduction in the boiling point. However, vacuum drying at high temperatures created voids on the surface of the pattern due to the solidified surface. AFM images of the inkjet-printed ink solidified in the vacuum condition in the microchannel at room temperature and at high temperature are provided in Figure 6c,d, respectively.

The residual solvent in the ink evaporated completely, and the roughness of the pattern was smooth for the room temperature condition. However, for the high-temperature condition, the residual solvent in ink was forced to evaporate through the solidified surface, and this created voids in the pattern. Therefore, the temperature condition in the vacuum drying process of the microchannel structure is a very important element in the formation of the pattern for the sublimation transfer process.

**Figure 7:**
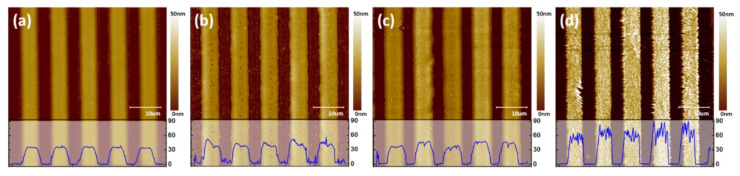
AFM images of sublimation transferred patterns after the drying process (**a**) at room temperature, (**b**) 30 °C, (**c**) 50 °C, and (**d**) 80 °C in a vacuum chamber. Insets are cross-sectional data, respectively.

**Figure 8:**
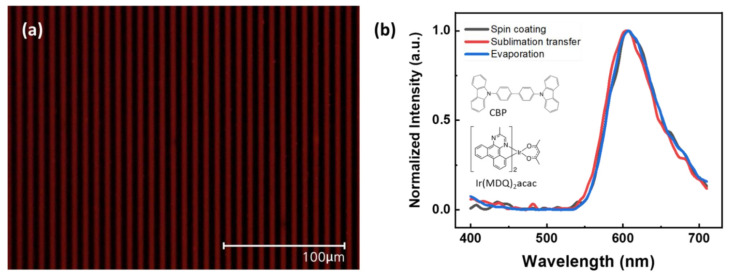
(**a**) OM image with UV exposure of sublimation transferred pattern. (**b**) Normalized PL spectra at an excitation wavelength of 365 nm, according to the patterning methods; sublimation transfer, evaporation, and spin-coating. Insets are the chemical structure of the emitting materials; CBP and Ir(MDQ)_2_acac.

AFM images of sublimation transferred patterns according to drying conditions are provided in Figure 7. Figure 7a shows a sublimation transferred pattern formed clearly from the dried donor substrate in the vacuum chamber at room temperature conditions. However, the sublimation transferred pattern under drying with heat in the vacuum condition is coarse and rough, and includes voids, as shown in Figure 7b–d. Additionally, with higher temperatures in the vacuum chamber, the sublimation transferred pattern had greater roughness. In addition, Figure S3a,b provide OM images of the sublimation transferred patterns that were dried in a vacuum and in the air atmosphere at room temperature, respectively. In the case of drying in the vacuum condition, a clear pattern was formed compared to that for the air atmosphere. This is due to the microchannel width of only about 4 μm, where residual solvents were trapped in this narrow gap. As a result, drying in the air atmosphere with heat causes the materials to break and separate out from the microchannel; without heat, this process induces voids in the inkjet-printed pattern. Drying in the vacuum condition with heat causes poor surface roughness of the pattern. On the other hand, drying in the vacuum condition without heating completely evaporates the residual solvents due to the reduction in the boiling temperature. These drying conditions influence the sublimation transferred pattern. Table S2 provides a summary of characteristics of the sublimation transferred patterns according to the dry conditions. Figure 8a provides a UV exposed OM image of the sublimation transferred pattern in a wide range of areas. RMS roughness of 1.331 nm was obtained on the top of the sublimation transferred pattern at room temperature in a vacuum, as shown in Figure S4. Additionally, the absorption spectrum of the Ir(MDQ)_2_acac and PL spectrum of the CBP was almost overlapped, suggesting effective energy transfer from CBP to Ir(MDQ)_2_acac molecules. The chemical structures of the emitting materials are provided in the inset of Figure 8b. Consequently, the emitting material has a dominant photoluminescence peak at 605 nm from the Ir(MDQ)_2_acac, which has been found in a number of studies [43,44]. Normalized PL spectra at an excitation wavelength of 365 nm for the various patterning methods for validation are provided in Figure 8b. The peak at around 605 nm from the red dopant was measured and found to have no wavelength shifts even when using the inkjet printing method grafted onto the sublimation transfer process; this is comparable to the case of the conventional thermal evaporation method.

## 4. Conclusions

In conclusion, a 2822 PPI resolution pattern for the EML of OLEDs was successfully implemented via inkjet printing grafted onto the sublimation transfer process. The developed co-solvented ink for inkjet printing was printed under optimized conditions of ink filling into the microchannel on an LTHC substrate. The microchannel and bank of the LTHC substrate turned into hydrophilic and hydrophobic, respectively, by selective surface treatment. The selective surface treatment easily induced ink into the microchannel via inkjet printing. The amount of ink that filled the microchannel was influenced by the drop space and line space. Inkjet-printing conditions for the microchannel were optimized at a value of 9 μm for line space and 25 μm for drop space, with a donor substrate of 2822 PPI. Furthermore, the drying condition after the inkjet printing process significantly influenced the sublimation transferred pattern formation and morphology. Subjecting the inkjet-printed donor substrate to a drying process with heat in a high vacuum chamber brought about rapid solidification of the induced ink surface, and voids are formed in the ink. On the other hand, drying the ink pattern at room temperature in a high vacuum eliminated the formation of void areas because of the rapid exhaustion of residual solvent before the surface dried completely. Finally, without any wavelength shifts, we successfully implemented a 2822 PPI resolution EML pattern for OLEDs by improving the inkjet printing and drying processes grafted onto the sublimation transfer process. In further studies, we will attempt to manufacture the pixel define layer for the OLED device as donor substrate resolution, and by using this, it will be fabricated into the OLED device. This suggests that inkjet printing technology will be sufficiently applicable to realize high-resolution EML patterns by applying our technology.

## Data Availability

The data presented in this study are available on request from the corresponding author.

## Supplementary Materials

The following supporting information can be downloaded at: https://www.mdpi.com/article/10.3390/nano12091611/s1, Figure S1: Printable region and drop speed of co-solvented inks by controlling pulse time and voltage of waveform; Figure S2: Optical microscope images of sublimation transferred patterns dried at 80 °C for 20 min in air atmosphere; Figure S3: Optical microscope images of sublimation transferred patterns with UV exposure according to dry conditions; Figure S4: AFM images of sublimation transferred patterns after drying process at room temperature that after flattening process; Table S1: Properties of organic solvents used to produce co-solvented ink; Table S2: Summary of results of inkjet-printed pattern in microchannel and sublimation transferred pattern according to the dry conditions. References [45,46,47] are cited in the Supplementary Materials.
