# Supplementary material for: High-Resolution Patterning of Organic Emitting-Layer by Using Inkjet Printing and Sublimation Transfer Process
Source: Nanomaterials (Basel). 2022 May 9;12(9):1611. doi: 10.3390/nano12091611 (PMC9100090; doi:10.3390/nano12091611)
Supplement: Supplementary file 1 [file nanomaterials-12-01611-s001.zip › nanomaterials-1694190-supplementary.pdf]

Table S1. Properties of organic solvents that are used for the production of co-solvented ink.

Solvents	Boiling Point (°C)	Viscosity = γ (g/cm s)	Surface Tension = μ (g/s²)	Density = ρ (g/cm³)	Vapor Pressure (cmHg)
**Chlorobenzene (CB)**[1]	131.7	0.0073	23.93	1.11	0.88
**1,2-Dichlorobenzene (oDCB)**[2]	180.5	0.01322	36.63	1.306	0.136
**N,N-dimethylformamide (DMF)**[3]	153	0.0092	36.5	0.948	0.4208

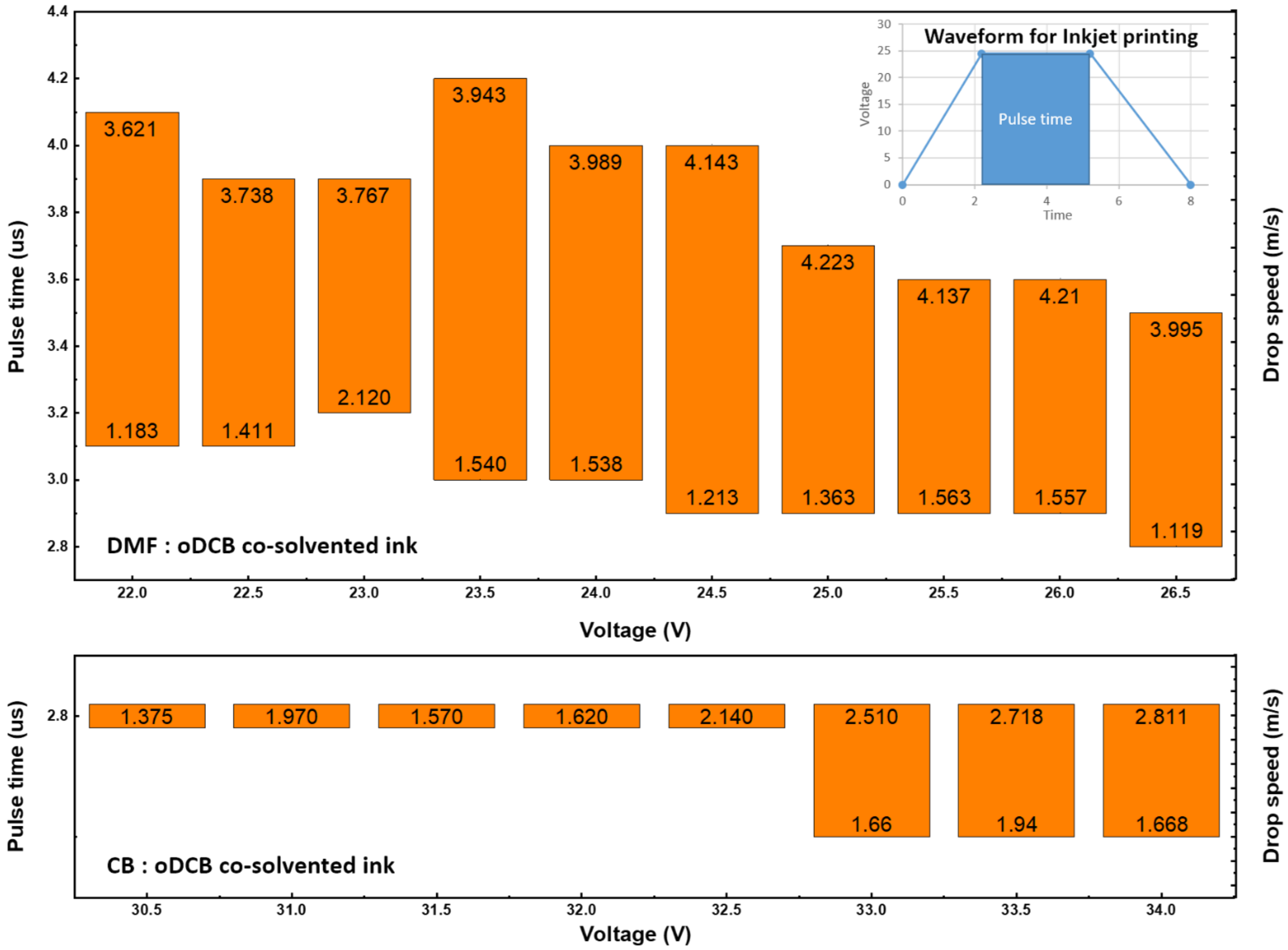

Figure S1. Printable region and drop speed of co-solvented inks by controlling the pulse time and voltage of the waveform.

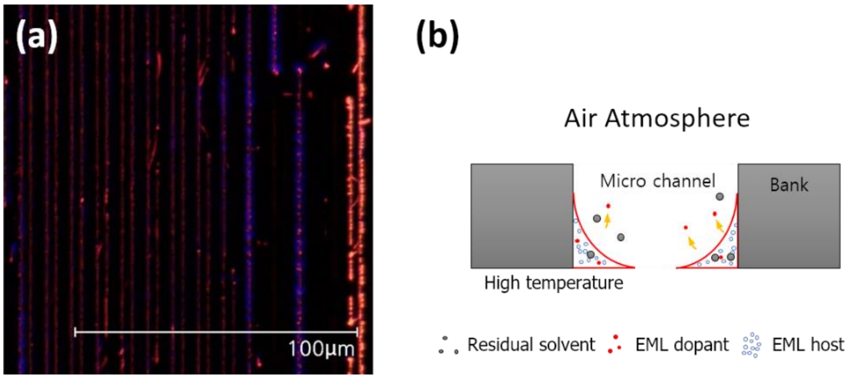

Figure S2. (**a**) Optical microscope images with UV exposure of inkjet-printed onto the microchannel that is drying in an air atmo phere at 80 °C for 20 minutes. (**b**) Schematic diagram of the residual solvents breaking out the pattern from the microchannel.

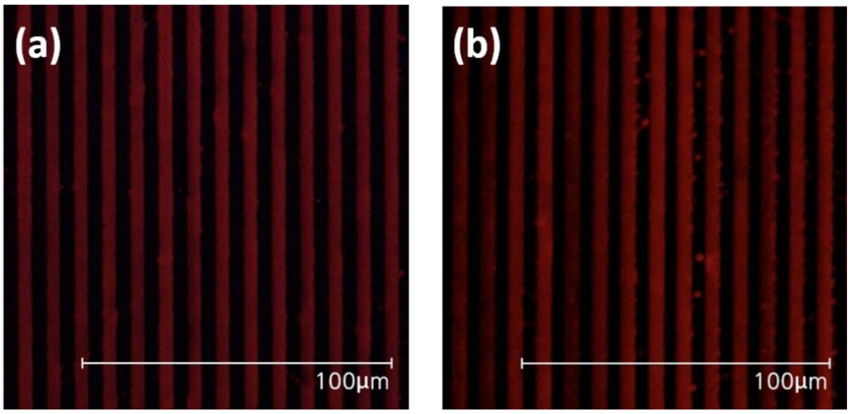

Figure S3. Optical microscope images of sublimation transferred patterns with UV exposure according to drying (**a**) in a vacuum chamber and (**b**) in an air atmosphere at room temperature.

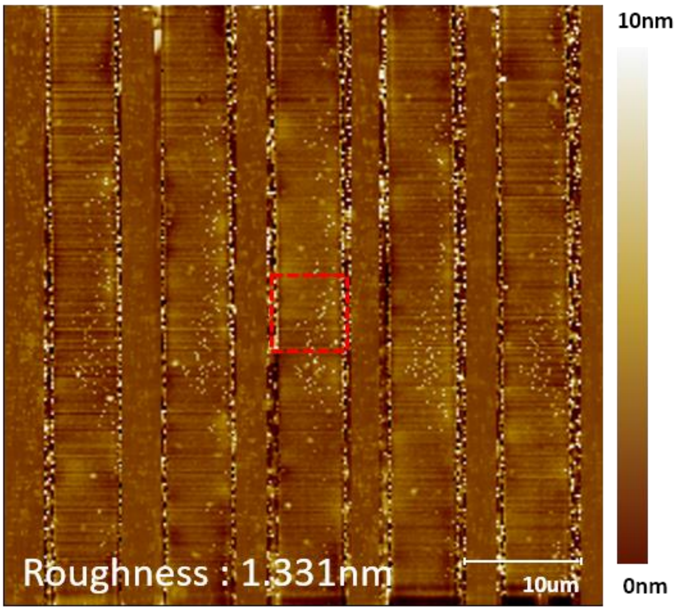

Figure S4. AFM images of sublimation transferred patterns after the drying process at room temperature after the flattening process. The marked area on the pattern was used for roughness calculation.

Table S2. Characteristics of the sublimation transferred patterns according to the dry conditions of inkjet-printed patterns in microchannel.

Drying Condition	Air atmosphere		Vacuum Condition	
	**Without Heating**	**With Heating**	**Without Heating**	**With Heating**
**Inkjet-printed in micro-channels**	Not fully evaporation of residual solvent	Break and separate out from the microchannel	Fully evaporation of residual solvent and having smooth roughness	Coarse surface roughness due to fast evaporation
**Sublimation transferred pattern**	Voids & coarse roughness	Pattern not formed	Fine patterns and smooth roughness	Voids & coarse roughness

References

1. Rankin, D.W.H. CRC Handbook of Chemistry and Physics, 89th Edition, Edited by David R. Lide. *Crystallography Reviews* **2009**, *15*, 223–224, doi:10.1080/08893110902764125.
2. National Center for Biotechnology Information (2022). PubChem Compound Summary for CID 7239, 1,2-Dichlorobenzene Available online: https://pubchem.ncbi.nlm.nih.gov/compound/1_2-Dichlorobenzene (accessed on 28 March 2022).
3. National Center for Biotechnology Information (2022). PubChem Compound Summary for CID 6228, N,N-Dimethylformamide Available online: https://pubchem.ncbi.nlm.nih.gov/compound/N_N-dimethylformamide (accessed on 28 March 2022).
